# Using the Tissue Impulse Response Function to Streamline Fractionated MRgFUS-Induced Hyperthermia

**DOI:** 10.3390/cancers17030515

**Published:** 2025-02-04

**Authors:** Pauline C. Guillemin, Yacine M’Rad, Giovanna Dipasquale, Orane Lorton, Vanessa Fleury, Shahan Momjian, Anna Borich, Lindsey A. Crowe, Thomas Zilli, Sana Boudabbous, Rares Salomir

**Affiliations:** 1 Image Guided Interventions Laboratory, GR-949, Faculty of Medicine, University of Geneva, 1205 Geneva, Switzerland; mrad.yacine@gmail.com (Y.M.); orane.lorton@hug.ch (O.L.); anna.borich@unige.ch (A.B.); thomas.zilli@eoc.ch (T.Z.); sana.boudabbous@hug.ch (S.B.); rares.salomir@unige.ch (R.S.); 2 Herosupport SA, Route de l’Uche 53, 1255 Veyrier, Switzerland; giovanna@herosupport.ch; 3 Clinical Neurosciences Department, University Hospitals of Geneva, Rue Gabrielle-Perret-Gentil 4, 1205 Geneva, Switzerland; vanessa.fleurynissen@hug.ch (V.F.); shahan.momjian@hug.ch (S.M.); 4 Faculty of Medicine, University of Geneva, 1205 Geneva, Switzerland; 5 Radiation Oncology, Oncology Institute of Southern Switzerland (IOSI), EOC, 6500 Bellinzona, Switzerland; lindsey.crowe@hug.ch; 6 Faculty of Biomedical Sciences, Università della Svizzera Italiana, 6900 Lugano, Switzerland; 7 Radiology Division, HUG, University Hospitals of Geneva, Rue Gabrielle-Perret-Gentil 4, 1205 Geneva, Switzerland

**Keywords:** hyperthermia therapy, HIFU, MRI

## Abstract

**Background/Objectives**: Combining radiation therapy with mild hyperthermia, especially via magnetic resonance-guided focused ultrasound (MRgFUS), holds promise for enhancing tumor control and alleviating symptoms in cancer patients. However, current clinical applications of MRgFUS focus primarily on ablative treatments, and using MRI guidance for each radiation session increases treatment costs and logistical demands. This study aimed to test a streamlined workflow for repeated hyperthermia treatments that reduces the need for continuous MRI monitoring, using an approach based on impulse response function (Green’s function) to optimize acoustic power settings in advance. **Methods**: We implemented the Green’s function approach in a perfused, tissue-mimicking phantom, conducting 30 experiments to simulate hyperthermia delivery via MRgFUS. Pre-calculated acoustic power settings were applied to maintain a stable hyperthermia target without the need for real-time feedback control from MRI thermometry. Additionally, a retrospective analysis of patient thermometry data from MRgFUS sonications was performed to assess feasibility in clinical contexts. **Results**: Our experiments demonstrated consistent, stable hyperthermia (+7 °C) for 15 min across varying perfusion rates, outperforming conventional closed-loop MRI feedback methods in maintaining temperature stability. The retrospective analysis confirmed that this method is noise-robust and clinically applicable. **Conclusions**: This off-line approach to hyperthermia control could simplify the integration of MRgFUS hyperthermia in cancer treatment, reducing costs and logistical barriers. These findings suggest that our method may enable the broader adoption of hyperthermia in radiation therapy, supporting its role as a viable adjuvant treatment in oncology.

## Simple Summary

This research explores a new method for combining radiation therapy with a special type of heat treatment (hyperthermia) aimed at making cancer treatments more effective. Probing tissues with focused ultrasound guided by MRI, we can subsequently deliver controlled, gentle heat to targeted areas without needing continuous MRI monitoring, which is often expensive and time-consuming. This study tested the method on tissue-like models and showed that it consistently reaches and maintains the desired temperature more reliably than traditional methods. This advance could make hyperthermia more accessible in cancer treatment by streamlining the process and reducing costs, offering a practical way to enhance the effects of radiation therapy. Ultimately, this approach may broaden hyperthermia’s use in hospitals, providing the research community and clinicians with a reliable, cost-effective tool for improving patient care in oncology.

## 1. Introduction

The integration of hyperthermia (HT) with radiation therapy (RT) represents a promising therapeutic approach in the oncological landscape, drawing upon established principles from radiobiology, molecular biology, and tumor physiology [1,2]. Hyperthermia, characterized by elevating the temperature of the targeted area to a range between 41° and 44° Celsius, holds substantial potential when combined with External Beam Radiation Therapy (EBRT). This synergy has been observed to potentiate cell killing and holds promise in mitigating adverse effects, potentially circumventing the need for escalated radiation doses or the use of systemic therapies in combination with EBRT [3,4,5,6]. Moreover, the augmentation of tumor oxygenation post-mild HT, lasting up to 24–48 h after treatment, has been associated with an increased favorable response to RT [7]. These compelling outcomes underscore the clinical significance of HT as an adjunct to conventional RT or innovative targeted radionuclide therapy [8], establishing it as an integral part of the therapeutic arsenal for cancer.

Radiotherapy and hyperthermia together have the potential to increase immune activity by releasing specific proteins and improving blood flow to the tumor. This treatment combination elicits a systemic anti-tumor immune response and triggers abscopal effects. Radiation induces the expression of pro-inflammatory cytokines, stimulates antigen presentation, and increases the sensitivity of tumor cells to immune responses. Hyperthermia has the potential to further amplify these effects by increasing immune recognition and T-cell trafficking, as well as promoting the secretion of chemokines [9]. Hyperthermia influences several molecular parameters involved in active radiosensitizing tumor cells and enhancing the potential of targeted radiotherapy. Notably, there is a large body of evidence suggesting that hyperthermia induces Hsp70 and HSPA1A synthesis and enhances telomerase activity [10].

While several technologies can be used to deliver heat into deep tissues, either by microwaves or radiofrequency with thermocouple sensors, focused ultrasound is the only non-invasive known technology that can enable sharply localized energy deposition inside deep tissues [11]. Notably, magnetic resonance-guided focused ultrasound (MRgFUS), employing modern phased array transducers, exhibits distinct advantages, including the accurate delineation of heating patterns, the customizable shaping of heating regions, and real-time temperature monitoring through advanced MR imaging sequences [12]. Its applications are expanding in both ablative treatments [13,14,15] and hyperthermia [16,17,18] across various tumor sites. It is clinically accepted for the thermal ablation of uterine fibroids [19,20,21], breast cancers [22,23], or prostate cancers [24], highlighting its versatility and potential in personalized oncology care [25,26,27,28,29].

However, a significant gap exists in current regulatory approvals in clinics, limiting focused ultrasound devices to ablative uses and lacking dedicated devices for hyperthermia applications. The setup, challenges, and workflow are significantly different between hyperthermia and thermal ablation. Despite challenges, several clinical studies have shown the various possibilities of hyperthermia by MRgFUS, such as conclusive results on the pain palliation of bone metastases [30,31,32,33] or its use to enhance nanomedicine delivery to solid tumors [34,35]. Nowadays, only one study has been published on patients for ultrasound-based hyperthermia adjuvant to radiation therapy [36]. That study concerns rectal cancer retreatment using concurrent chemoradiation and mild hyperthermia with MRgFUS. The results show that they were able to successfully treat patients with locally recurrent unresectable rectal cancer by combining MRgFUS mild hyperthermia, re-irradiation, and chemotherapy without unacceptable toxicity. Previously, Guillemin et al. [37] introduced a novel concept of extracorporeal phased array transducer designed especially for deep hyperthermia treatment. Using MR thermometry, we demonstrated that the phased array transducer allowed the precise delivery of hyperthermia inside the target volume, ensuring a fast patient setup by means of a transducer holder.

RT is generally delivered with fractionated protocols. Employing MRI-guided procedures before every fraction would significantly increase the costs and logistics. We thought to streamline the hyperthermia procedure, making it more accessible across diverse radiotherapy settings [38]. We aimed to validate a novel workflow where safe and accurate ultrasound-based HT is delivered without systematic MRI control. Reproducible targeting using immobilization devices has been described in a previous article by Guillemin et al. [39].

Here, we introduce the concept of performing only the first session of HT under MR control to compute the thermal Green’s function of the treated tissue. In brief, Green’s function is an integral kernel that can be used to solve some classes of inhomogeneous partial differential equations with boundary conditions. Back in the 1990s, a theory for solving the bioheat equation was developed using the time-dependent Green’s function and Fourier transform techniques [40]. The theory was illustrated for a thermal conduction hyperthermia system and a radiofrequency interstitial system. Later, the Green’s function approach was used to predict local RF heating in interventional MRI [41]. Furthermore, the possibility of measuring blood perfusion and thermal conductivity using the steady-state temperature distribution of a point source was also proposed [42].

The numerical Green’s function method in the context of MRgFUS has been used to generate temperature maps with high accuracy and determine in situ the Specific Absorption Rate (SAR) (the locally deposited thermal energy), minimizing the effect of measurement noise [43]. Moreover, a new MR shear wave elastography technique—using transient acoustic radiation force pulses from a focused ultrasound transducer—and a coding strategy based on Green’s function were used to assess the feasibility of calculating shear wave velocity maps using the analytical expression [44].

Besides the exploitation of Green’s function for the improved monitoring of SAR, Magnetic Resonance Thermometry, and Magnetic Resonance Acoustic Radiation Force (impulse) Imaging, we are suggesting here to use this information to compute off-line the prescribed acoustic power for subsequent long hyperthermia and deliver it per need outside the MR room, under the hypothesis of a nearly linear system. In addition, we have taken advantage of the present study to validate, based on a perfused model, the novel concept of hyperthermia HIFU transducer recently reported by Guillemin et al. [37].

## 2. Material and Methods

### 2.1. Theory

The so-called Green’s function is the impulse response of an inhomogenous linear differential operator defined on a domain with specified initial conditions and/or boundary conditions, when the right term (or the excitation) is the Dirac delta function. Theoretically, determining the Green’s function of a linear system is sufficient to calculate its response to any external excitation via a convolution process, and this also includes the effect of border conditions.

We aim to adapt and implement this formalism for a novel approach to the off-line control of MRgFUS hyperthermia. First, we are explicitly determining the voxel-specific Green’s function in the time domain. Second, we are solving the inverse problem for the hyperthermia acoustic power to be applied for a long High-Intensity Focused Ultrasound (HIFU) sonication, that is, the deconvolution between the target temperature curve at the focal point and that Green’s function. The theoretical Equation (1) is straightforward, using a 1D numerical Fourier transform in the temporal dimension, for instance, at the focal point location. However, the experimental and numerical implementations require specific developments as detailed in this report.

(1) $$prescribed\_power=iFFT\left[\frac{FFT\left(prescribed\_temperature\right)}{FFT(impulse\_response)}\right]$$

where FFT is Fast Fourier Transformation and iFFT is inverse Fast Fourier Transformation. Hardware and biophysical limitations lead to the impossibility of generating a Dirac delta sonication. An alternative solution is suggested here using a Gaussian-shaped excitation. The Gaussian function has several advantages for the Fourier transformation formalism. Its Fourier spectrum is also Gaussian, therefore, monomodal and ensuring a much faster monotonic convergence to zero compared to exponential decay, while avoiding zero crossings.

The following steps were designed, implemented, and validated in terms of numerical robustness:

- The excitation of the system with a Gaussian input;
- Recording and denoising the MR thermometry data for this quasi-impulse response;
- The deconvolution of the quasi-impulse temperature response and the Gaussian acoustic input;
- The truncation of the 1D Fourier spectrum;
- Filtering out the truncation-related oscillations of Green’s function;
- The deconvolution of the target temperature elevation and Green’s function to obtain the prescribed acoustic power for steady-state long hyperthermia.

Every spectrum in this work is a complex number vector and, for graphical illustration, its magnitude was plotted as a real number vector. Further insights into the numerical implementation are provided below.

**Figure 1:**
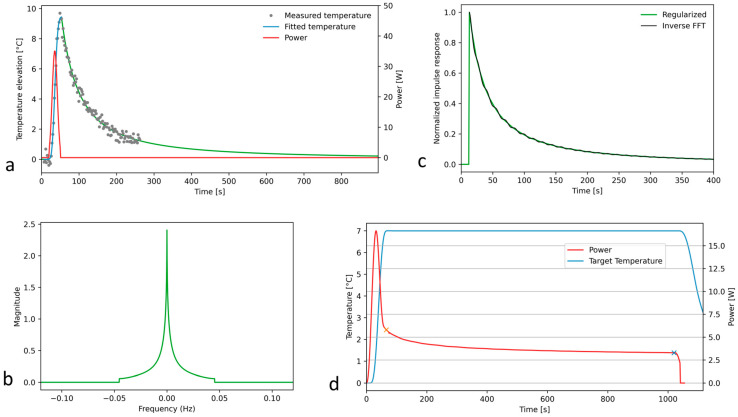
Numerical pipeline of the off-line computing of the impulse response function and the acoustic power for HIFU hyperthermia using illustrative MRgFUS experimental data. (**a**) Denoising of the experimental quasi-impulse response that was measured after a Gaussian excitation and extrapolated until approaching the zero value; (**b**) truncated 1D Fourier spectra of the impulse response function; (**c**) insight of the truncation-related oscillations of the impulse response function and their removal by searching the local inflexion points; (**d**) calculated acoustic power that should yield a rapid elevation of temperature and a further steady-state plateau for hyperthermia, when the prescribed power smoothly decrease from 5.8 [W] to 3.3 [W].

The MR thermometry data recording the system quasi-impulse response need a high sampling rate and spatial resolution, and, therefore, is intrinsically subject to noise. The first step was the denoising of the experimental quasi-impulse response. We used a model fitting curve as illustrated in Figure 1a, separately for the active heating (Equation (2)) and passive cooling (Equation (3)) regimens. The two models were connected as a continuous function at the point of temperature decrease of 10% below the maximum.

(2) $$∆T\left(t\right)={∆T}_{max}\cdot exp\left(-\alpha \cdot {\left|t-t_{0}\right|}^{\beta }\right)$$

The temperature elevation during the application of the Gaussian excitation (active heating regimen) was modeled by a generalized Gaussian function (GGF). GGFs are used in the engineering community for modeling many physical phenomena [45]. Here, assuming the impulse response function of our system is an ideal delta Dirac function, then the GGF response to the Gaussian excitation reduces to a common Gaussian function too, that is, β=2 in Equation (2). This model therefore satisfies the ideal limiting case.

(3) $$∆T^{\prime }\left(t\right)=\frac{∆T_{0}}{{\left[1+a\cdot \left(t-t_{0}^{\prime }\right)\right]}^{b}}$$

Equation (3) of the passive cooling regimen was derived from the analytical Green function of the heat equation [46] by generalizing the exponent of the denominator from 0.5 into a positive real value. This enables the model to adapt to other tissue parameters (e.g., perfusion) without adding extra factors to the equation, which would otherwise render the fitting ill-conditioned and, thus, unstable.

The fit of the cooling curve also enabled the analytical extension of the quasi-impulse response versus time until an arbitrary future point, practically until the end point of the planned steady-state hyperthermia. The model-adjusted, noise-free quasi-impulse response was further directly Fourier transformed and divided by the Fourier transform of the Gaussian input. According to the convolution theorem, this yields the spectrum of Green’s function. To avoid numerical instabilities, a cutoff was implemented at the position of the first local minimum of the spectral division, as shown in Figure 1b. The truncated spectrum was inverse Fourier transformed (Figure 1c), delivering the reconstructed time-domain Green’s function, showing minor oscillations. The latter ones were locally removed by determining the null values of the second-order derivative (inflexion points) and connecting them by linear segments. This filtered Green’s function was finally proportionally rescaled to satisfy the conservation of the energy, meaning that the area under the curve (AUC) of the quasi-impulse response should equal the product of the AUC of Green’s function by the AUC of the Gaussian excitation.

The convolution theorem was again applied to (i) the target temperature for hyperthermia and (ii) the time-domain Green’s function, to finally output the calculated time-domain acoustic power for HIFU hyperthermia (Figure 1d). Note that the filtered Green’s function spectrum is a smooth monomodal function without zero crossing points and was therefore not truncated for this last step.

The quality assurance of the numerical pipeline was performed by convoluting the time-domain acoustic power with the tissue impulse response function, to verify the result is identical to the predefined target temperature for hyperthermia.

### 2.2. Perfused Phantom

**Figure 2:**
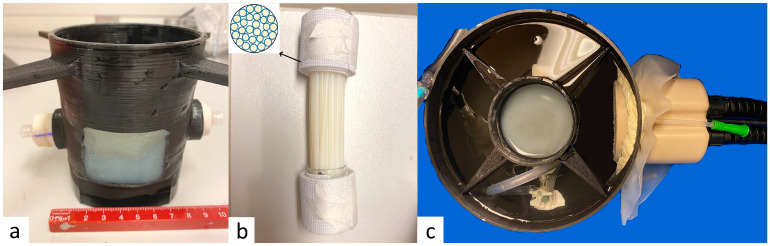
(**a**) The multi-compartmental perfused phantom model, embedding the matrix gel and the interstitial gel-filled PTFE capillaries. Note the rectangular acoustic window. (**b**) Multiple PTFE capillaries, densely packed, encapsulating a tissue-mimicking gel. (**c**) The full setup connected to a perfusion machine and aligned with the HIFU transducer, securely fixed in place.

A multi-compartmental perfused phantom model has been developed, featuring a 3D-printed structure perforated to provide three outlets, one rectangular outlet for an ultrasound access window (45 mm × 35 mm) plus two circular outlets 22 mm in diameter positioned face-to-face, aligned with a cylindrical cavity of 22 mm designed to house a 20 mL plastic syringe. This was filled with a matrix comprising 2% agar (Figure 2a). The agar-based matrix provides geometrical structure and non-attenuated ultrasound propagation and drives the perfusion fluid flow. Additionally, a second gel, possessing ultrasound sensitivity closely resembling biological tissue, was crafted using degassed water, 11% *w*/*w* glycerol (Acros Organics, Thermo Fischer Scientific, Geel, Belgium), 10% *w*/*w* powdered milk (8.5% fat, 7.1% proteins), and 3% *w*/*w* agar (Alfa Aesar, Karlsruhe, Germany). This gel was used to fill 52 capillaries made of PTFE (polytetrafluoroethylene), with a wall thickness of 0.2 mm, inner diameter of 1.8 mm, and length 80 of mm. The PFTE material has a high ultrasound absorption (a = 0.144 dB.${mm}^{-1}$.${MHz}^{-1}$) [47], more than twice the value of ultrasound absorption by muscle [48] and is therefore the main source of heat generation in this system, guaranteeing the reproducibility of the results. The bundle of PFTE capillaries was densely packed (Figure 2b) and inserted into the 22 mm cylindrical cavity of the matrix gel, yielding an approximately 90% volume filling ratio. To prevent the introduction of air bubbles into the phantom, this process is conducted with the phantom immersed in degassed water. The presence of trapped air bubbles can potentially create barriers for HIFU and lead to susceptibility artifacts on MR images.

This geometric arrangement replicates the tissue structure irrigated by blood flow. The model was perfused using a tunable flow rate and MR-compatible perfusion machine, which comprised a drive module with the automatic feedback control of pressure, an ‘umbilical cord’ connecting the drive module, and a perfusion module as described by Buchs et al. [49]. We connected the perfusion machine to the described tissue-mimicking model and aligned the entry window with the HIFU beam (Figure 2c) of a phased array transducer, as described below. To validate the model’s reliability, we monitored the perfusion between the capillaries using Doppler ultrasound imaging (similar to Lorton et al. [50]) and an MRI flow sequence (similar to Holman et al. [51]).

**Figure 3:**
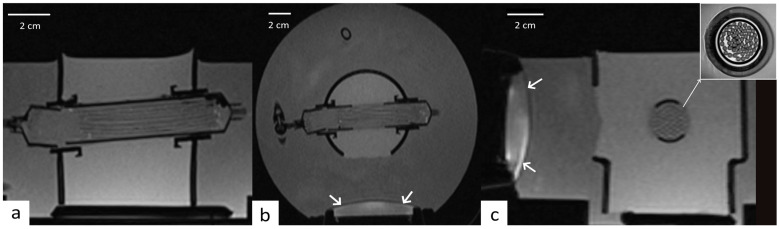
Three-dimensional MR targeting based on VIBE acquisition; three orthogonal planes are shown ((**a**) axial, (**b**) coronal, and (**c**) sagittal). Note the high-resolution T2 TSE inset orthogonal to the gel-filled PFTE capillaries on (**c**), and the water-cooling space in front of the transducer (white arrows).

During the experiments performed under 3T MRI guidance (Prisma Fit, Siemens, Erlangen, Germany), the data were acquired with a combination of a standard spine coil and a flexible surface coil. The geometry of perfused phantom and the position of HIFU transducer was verified on a high-resolution T1-weighted 3D Gradient Echo (GRE) (VIBE, Repetition Time (TR): 5.44 ms, Echo Time (TE): 1.81 ms, flip angle: 10, Bandwidth (BW): 390 Hz, GRAPPA = 2, PF = 0.75 × 0.75, Acquisition Time (TA): 2 min 54 s, voxel size 0.8 mm × 0.8 mm × 1.2 mm, slice direction LR, Field of View (FOV) = 256 × 256 × 268 mm^3^). These images were also used to confirm the degassing of the perfused model, that is, the suppression of macroscopic air bubbles in the interstitial compartment (Figure 3a,b). The high-resolution T2-Turbon Spin Echo (TR: 3000 ms, TE: 44 ms, flip angle: 160°, BW: 160 Hz, TA: 9 min 15 s, 0.2 mm × 0.2 mm × 5 mm) allowed the visualization of the regular packing of strands and the evaluation of the volume filling ratio of tissue/fluid at approximatively 9:1 (Figure 3c).

### 2.3. MRI Guidance of Local Hyperthermia

The temperature elevation (ΔT) in tissue-mimicking gel was determined through the application of the proton resonance frequency shift (PRFS) method. This method relies on analyzing temperature-induced alterations in the phase of the gradient echo signal. The data were acquired using interleaved, segmented echo-planar imaging (EPI) with a factor of 7, ensuring a temporal resolution of 2.7 s. The imaging parameters included a FOV of 192 × 192 mm^2^, a slice thickness of 4 mm, and an in-plane resolution of 1.5 mm. Further parameters were the following: TR: 50 ms, TE: 10 ms, flip angle: 20°, BW: 164 Hz, EPI factor of 7, and spectral-selective fat suppression.

### 2.4. Focused Ultrasound

Focused ultrasound was generated by an MR-compatible 192-element phased array transducer (Imasonic, Besançon, France), designed specifically for deep local hyperthermia. Elements are pseudo-randomly distributed in 6 columns based on a cylindrical surface, with an average active surface of 1670 mm^2^. The frequency of the ultrasound was 700 kHz. Further details are provided in Guillemin et al. [37]. The transducer was powered by a multi-channel beam former (10 W/channel, 256 channels, independent control of phase and amplitude) manufactured by Image Guided Therapy (Pessac, France). The procedure was controlled by a home-written multi-thread application using Python (Python Software, version 3.7, Beaverton, OR, USA) for Windows 7 and 10. The real-time control of the power beam former was implemented using the manufacturer’s drivers (“IGTFUS” library). The quality assurance for the execution over a long duration (up to 20 min) of predefined acoustic parameters was implemented by re-applying the device-logged information from previous sonication and comparing the MR temperature data under identical conditions.

### 2.5. Experimental Procedure on Perfused Phantom

Following a period of thermal stabilization at room temperature (22 °C) and degassing under vacuum for 30 min, a perfused phantom was immersed in a water-filled bath that was acoustically coupled with the HIFU transducer (refer to Figure 2c).

The perfusion was regulated, and the total flow rate through the phantom was set in the range of 2.8 mL/min to 5.6 mL/min, yielding a comparable flow density to prostate perfusion in humans [52].

To calculate the power required for achieving a specific target temperature profile, the approach described in Section 1) was employed, using the Gaussian excitation profile and Fourier analysis. This process began with the application of a Gaussian profile of acoustic power, a peak value of 30 W to 40 W, a duration of 30 s, sampling steps = 20, and FWH = 3 s. This is a pseudo-Dirac excitation employed to induce temperature variations within the system with an increase in temperature to the order of +10 °C, thus avoiding the risk of irreversible thermal damage. Unlike a Dirac pulse, which is ideal but impractical due to the high risk of transducer damage and the lack of temperature elevation control, the Gaussian profile sonication was compatible with the device performance, being designed to be as brief as possible while enabling the safe and robust measurement of the quasi-impulse response function.

After the application of the Gaussian excitation, home-written Matlab R2021a (MathWorks, Natick, MA, USA) code was used to calculate and denoise the temperature elevation in the pixel of interest. Home-written Python (Python 3 Reference Manual. Scotts Valley, CA: CreateSpace) code was used for the remaining computing steps, driving the hardware and the writing of the sonication configuration files.

We conducted 10 Gaussian excitations for each level of perfusion (without perfusion, with low perfusion (4.4 mL/min), and with higher perfusion (5.6 mL/min)) and performed the subsequent 30 experimental MRgHIFU hyperthermia under on-line MR Thermometry while executing the pre-calculated sonication, aiming to deliver a temperature elevation of 7 °C for 15 min. This temperature elevation corresponds to 44 °C in patients, that is, the upper meaningful level of oncologic hyperthermia. Therefore, we investigated the current approach under the most demanding conditions. Preliminary experiments with our setup showed that extending the HT duration beyond 15 min did not provide additional information on the system behavior.

To investigate the effect of deviations from linearity and to replicate biological phenomena, such as temperature-dependent blood vessel dilation, repeated “stress test” experiments (n = 3) were performed with a dynamic change in perfusion rate during the application of hyperthermia. The perfusion rate started at 2.8 mL/min, was abruptly increased at 5.6 ml/min after 500 s of hyperthermia, and further maintained at this value until the end point of 900 s. In return, the pre-calculated curve of acoustic power, determined by Green’s approach for a fixed perfusion rate of 2.8 mL/min, was not modified (that is, it was executed without knowledge of perfusion change).

### 2.6. Comparison Experiments

In order to check the robustness and reliability of our method, we compared it with the previously reported predictive temperature controller for hyperthermia [53], which automatically adjusted the acoustic energy deposition on-line based on a conventional closed-loop implementation.

Consequently, we conducted a total of 15 comparative experiments, closed-loop feedback versus off-line pre-calculated acoustic power using the Green’s function formalism, under identical setup conditions (e.g., focal point position and perfusion flow rate).

### 2.7. Retrospective Analysis of MRgFUS Thalamotomy Data

The Green’s function algorithm was retrospectively applied to 2 essential tremor patients who underwent clinical routine MRgFUS thalamotomy to ensure the in vivo feasibility. Written informed consent to reuse their intervention data was obtained from both patients. The patients were treated by MRgFUS using the ExAblate 4000 device v1.1 (Insightec, Haifa, Israel) [50]. The target for the ablation was the ventralis intermediate nucleus (VIM) in the thalamus. The device, working at 670 kHz, is composed of 1024 elements distributed on a hemispherical helmet. For patient A, the data used comprised the last consolidation sonication, meaning that the perfusion was already locally interrupted. For patient B, the data used in the study comprised the first sonication reaching ablative temperature, immediately after the usual low-energy verification shots. The required energy for both cases was much higher than on the ex vivo sample, as a high portion of ultrasound is absorbed and reflected when passing through the skull. Technical information regarding the sonication is reported in Table 1. The temperature monitoring was performed under MR thermometry in the axial or coronal plane (FLASH GRE, TR/TE = 30.28/15.91 ms, slice thickness of 3 mm, in-plane resolution of 1.1 mm, temporal resolution of 3.9 s). The MR follow-up on the day after intervention confirmed the ablation at the expected target in the 2 patients. Using these data, we retrospectively calculated the theoretical power to apply to maintain a stable temperature of virtual hyperthermia treatment.

## 3. Results

### 3.1. Off-Line Hyperthermia Control in Perfused Phantoms

Experiments on perfused phantoms successfully demonstrated the technical feasibility of our protocol. No interference was detected between the MR scanner and the therapeutic ultrasound device.

**Figure 4:**
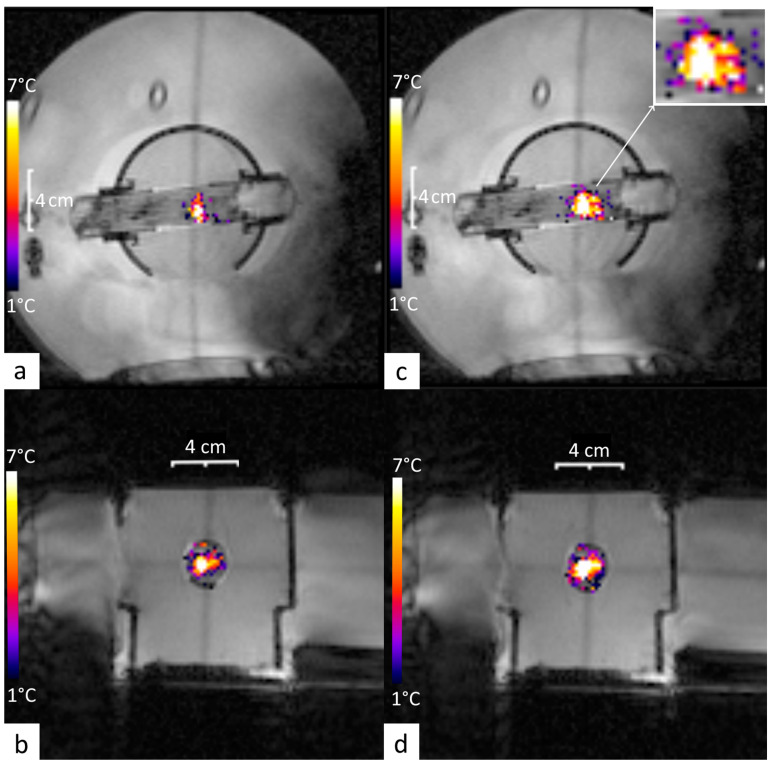
MR images of the gel phantom merged with PRFS thermal maps in coronal and sagittal planes during a 15 min HIFU sonication. (**a**,**b**) are captured at the time to steady-state (TtoSS: time to reach 90% of the desired temperature) that occurs at 56.7 s. (**c**,**d**) are captured at 7 min, representing half of the total duration of hyperthermia. Note the embedded distance scale and the temperature elevation color bar. Zoom inset: the drift of the thermal buildup along the direction of the fluid flow (here, from left to right).

**Figure 5:**
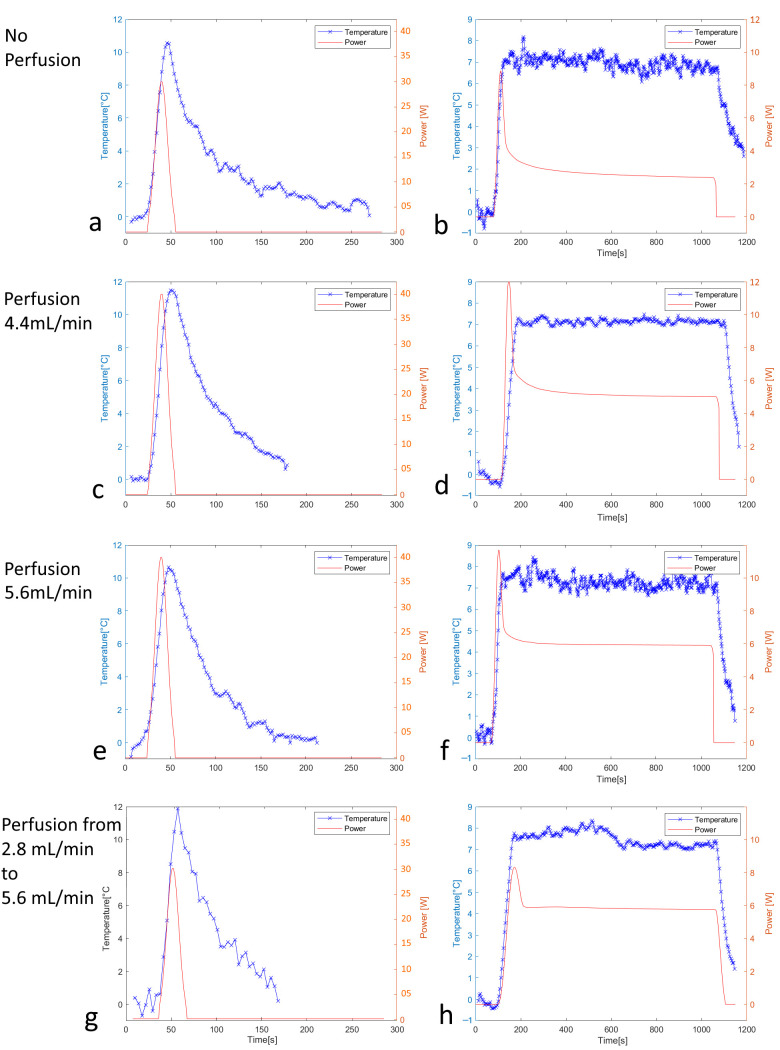
(**a**,**c**,**e**,**g**) Quasi-impulse response function in perfused gel phantom according to the flow rate conditions indicated on the side. (**b**,**d**,**f**,**h**) The respective experimental temperature (blue curve) versus time evolution during long sonications, using the pre-calculated acoustic power (red curve) based on Green’s function formalism. Note that the perfusion rate was deliberately doubled at t = 500 s for the plot h) to evaluate the influence of non-linear conditions.

The geometric targeting was accurately achieved on the central section of the perfused phantom, as shown in the MRI images merged with PRFS thermal maps in Figure 4. Moreover, the post-processing of PRFS thermometry data showed stable hyperthermia for each perfusion level (average temporal drift inferior to 10^−3^ °C/s) in tissue-mimicking phantoms at +7 °C, over a duration of 15 min, as described in Table 2 and Figure 5. Additionally, the time to reach 90% of the desired temperature was remarkably short, even with perfusion (under 59.4 s).

The higher the perfusion, the higher the calculated and applied acoustic power as shown in Figure 5. Also, the steady-state temperature regimen required approximately constant power when the tissue was, as shown in perfused Figure 5d,f, unlike gradually stepping down the applied power in the absence of perfusion as in Figure 5a.

Experiments (n = 3) with a dynamic change in perfusion demonstrated that doubling the flow in the course of application of the pre-calculated power resulted in a limited offset in the temperature evolution of approximately 10% (Figure 5h). This suggests that temperature-induced changes in tissue perfusion [54,55,56] would not impact the treatment benefit, which requires keeping the absolute temperature in the range of 41 °C to 43 °C.

### 3.2. Conventional On-Line Control Loop Versus the New Approach Using the Impulse Response Formalism

Experiments on a perfused phantom have shown that the on-line feedback control algorithm succeeds in maintaining a temperature of around 7 °C, but, as can be seen in the comparison of Table 2 and Table 3, the novel approach outperformed the conventional feedback control for the current conditions. Indeed, the temperature fluctuations during the steady-state regimen improved from 0.8 °C achieved with conventional feedback to 0.3 °C using the impulse response formalism. Moreover, the temporal average drift is one order of magnitude higher with the feedback technique (7.8 × 10^−3^ °C/s) than with the impulse response formalism (0.7 × 10^−3^ °C/s). Finally, the time to steady-state (TtoSS) is threefold higher with the feedback technique (150 s) than with the current one (50 s). These improvements can be ascribed to two factors: first, the a priori knowledge of the local tissue behavior via its impulse response function, and second, the denoising capability inherent in our mathematical model fitting analytical functions on experimental data.

### 3.3. Retrospective Analysis of Clinical MRgFUS VIM–Thalamotomy Data

**Figure 6:**
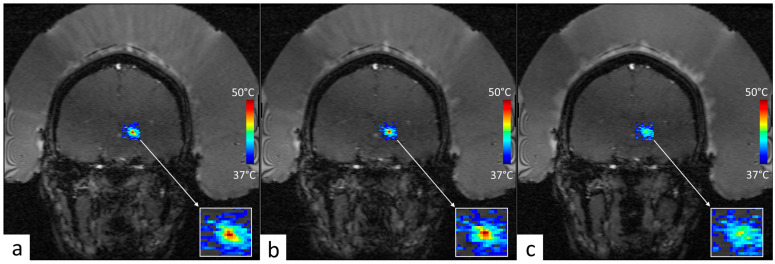
Near-real-time PRFS MR thermometry overlaid on GRE magnitude data for the MRgFUS sonication in patient #1. The FOV is 280 mm square and the temperature colormap ranges from physiological baseline (37 °C) to absolute 50 °C. (**a**) shows 4 s before the end of sonication, (**b**) shows the end of sonication, and (**c**) shows 4 s after the end of sonication; see acoustic parameters in Table 1. Note the acoustic streaming visible in the water layer between the head and the hemispherical transducer.

**Figure 7:**
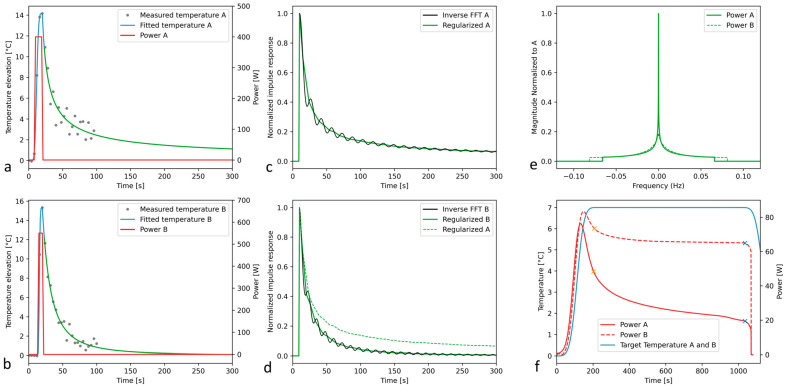
Extraction of the Green’s function of sonicated loci in two patients undergoing MRgFUS thalamotomy and its virtual exploitation to preset a steady-state hyperthermia sonication. (**a**,**b**) show the denoising and extrapolation of the quasi-impulse response function as obtained after a trapezoidal profile of acoustic power. (**c**,**d**) show the reconstructed temporal Green’s function for the two patients; note the significant offset between the curves, suggesting a clear difference in tissue perfusion. (**e**) shows the two spectra of Green’s functions normalized to the highest, and (**f**) shows the calculated acoustic power that should be theoretically required to maintain a steady-state plateau of +7 °C for each case.

Our numerical pipeline demonstrated stable performance under physical “stress” conditions, in particular temperature data generated with the tunnel coil (low SNR) and very short sonications (8 to 12 s) approaching a delta Dirac excitation, with only two or three sampled points of MR thermometry during the sonication itself (Figure 6). While the temperature data were acquired using the tunnel coil, in the presence of the HIFU helmet, the fit-based denoising worked accurately. The computing of the thermoacoustic Green’s function yielded a clear distinction between patient cases A and B, in terms of local perfusion, as shown in Figure 7. Case A, being a control sonication for the quality assurance of the actual focal point coordinates, was performed in already ablated tissue; its perfusion effect was very weak, unlike in case B, where previously non-treated tissue was sonicated. This difference is apparent for the cooling down rates (Figure 7d), as well as for the Green’s function spectra (Figure 7e) and the virtual acoustic power curves required to maintain a temperature plateau during long-lasting mild hyperthermia at that location (Figure 7f). The required power would fall from 48.3 W to 19.4 W (60% change) in the poorly perfused tissue, while it would fall from 73.3 W to 64.5 W (12% change) in the normally perfused tissue.

## 4. Discussion

The integration of hyperthermia via magnetic resonance-guided focused ultrasound (MRgFUS) with radiation therapy (HT-RT) presents a promising avenue in oncology, addressing a critical need for enhanced therapeutic options. For instance, Kok et al. estimated that the addition of HT provides an equivalent delivered dose of 10 Gy higher than RT alone [57]. Our study aims to contribute to this evolving landscape by developing a novel workflow, a pathway towards more accessible and cost-effective ultrasound-based hyperthermia, potentially leading to broader adoption in radiotherapy settings.

Our study demonstrates a robust, off-line-controlled hyperthermia workflow using MRgFUS, achieving precise and sustained temperature elevation, +7 °C for 15 min, in perfused phantom models without continuous MRI guidance. The validation experiments demonstrated consistent temperature elevations outperforming the conventional on-line closed-loop temperature control. Additionally, the temperature was accurately driven to the steady-state plateau much faster than with the conventional feedback method. While an extracorporeal HIFU device was used here, the method may be further adapted to local or interstitial applicators of therapeutic ultrasound [14,58].

The perfused phantom model employed in our study, featuring PTFE capillaries filled with tissue-mimicking gel, was designed to replicate the complex, inhomogeneous nature of human tissue. While this inhomogeneity can introduce variability in the thermal response, the heat conduction mitigates these variations at the observation scale to the order of 1 mm. It acts as a spatially smoothing effect of temperature gradients with time. Moreover, the use of PTFE (polytetrafluoroethylene) capillaries in our phantom model offers several distinct advantages. PTFE’s inert nature and low friction coefficient ensure minimal interaction with the perfusion fluid, preserving the flow dynamics that closely mimic biological tissue. Additionally, the uniform and constant acoustic absorption of PFTE material, which was the main source of heat generation in this system, together with the standardized diameter and regular distribution of these capillaries, yielded a reproducible model for studying perfusion effects on hyperthermia, unlike the plain soft gel strands reported by Lorton and Holman [50,51]. The material’s stability under repeated heating cycles further enhances the model’s reliability, allowing for accurate and repeatable measurements essential for validating our proposed workflow. This perfused model may therefore be used as a quality tool before each patient session, enabling us to ensure targeting quality (operation of all transducer elements) and the correct detection of power delivery by our software.

The foreseen field of application is adjuvant hyperthermia in oncology, dealing with low-temperature elevation (4 to 6 °C) not inducing structural changes in tissue, unlike ablative methods aiming for irreversible lesions in tissue. We, therefore, hypothesize for this range of temperature elevation that the deviation of the system from linearity is weak. In other words, the biological non-linear system (e.g., temperature-dependent perfusion rate) is still correctly approximated by a linear system, and we can apply Green’s function formalism with good accuracy. Of interest, the living tissue perfusion may change as a consequence of temperature elevation during the hyperthermia regimen [59]. Blood circulation through the heated tissue locus may gradually be accelerated due to heat-induced vasodilation, thereby decreasing the expected temperature. Therefore, we tested the current approach with a dynamic change in perfusion rate in vitro, while not adapting the pre-calculated acoustic power. Unlike the regimen of ablative MRgHIFU emphasizing rapid temperature variations, we are addressing here long hyperthermia delivery under steady-state conditions. Accordingly, temperature gradients are less steep, and fresh blood has more time to approach or reach thermal equilibrium with heated tissue. Overall, doubling the perfusion has shifted down the plateau of temperature by approximately 10%. This would definitely be acceptable under clinical conditions.

The impulse response function implicitly contains the information on acoustic absorption, heat diffusion, and blood perfusion at the respective location, under the approximation of a linear system. While these parameters are not explicitly extracted by our method, the computed power profile of acoustic power, aiming to maintain a constant temperature, reacts to the local perfusion; see, for instance, the different behavior of the plots in Figure 7f.

On top of the described off-line temperature control algorithm, aiming to avoid the need for on-line MRI guidance at every session, our approach for fractionated RT also requires the reproducible positioning of the extracorporeal HIFU transducer with respect to the defined target. One solution was described by Guillemin et al. [39] as consisting of an MR-compatible, 3D-printed, patient-specific immobilization device, embedding a holder for imaging or therapeutic ultrasound applicator. The 3D-printed device demonstrated sub-millimetric accuracy and ensured a fast patient setup and an optimal inter- and intra-fractional suppression of motion.

However, it is essential to acknowledge the limitations of our study, including its reliance on perfused phantom models and the need for further validation in clinical settings. While the phantom model used in our study offers a controlled and reproducible environment for validating the proposed protocol, including the dynamic adjustment of the perfusion rate, it has inherent limitations that must be acknowledged when considering its application to more complex tissue types. Unlike real tissues, the phantom lacks the intricate vascular architecture characteristic of living organisms. In vivo tissues exhibit spatially varying acoustic and thermal properties, such as ultrasound absorption and heat conduction coefficients, which can be affected by factors like fat content, fibrosis, or tumor composition. Despite these limitations, the phantom model remains an essential tool for preliminary evaluation, providing a simplified yet effective framework to test the feasibility and robustness of the proposed workflow in a perfused heterogeneous medium as visible in Figure 3c (inset).

If this technology is to be applied in clinical settings, several challenges need to be considered. First, in this initial study, the method was applied with fixed focal point sonication corresponding to a spatial scale of the thermal buildup to the order of 1 cm; see Figure 4. Extension to a pattern of foci covering a larger volume should consider the longer cooling time from such a thermal buildup, and, therefore, a longer time of acquisition to record the relaxation of the quasi-impulse response of temperature. Second, the variability in patient anatomy, tissue composition, and perfusion rates may necessitate the spatial mapping of the impulse response function with multiple independent sonications, impacting the total duration of the procedure. Therefore, future research should focus on optimizing treatment protocols, refining the measurement of Green’s function in vivo, and conducting clinical trials to evaluate the long-term safety and efficacy of this novel approach to ultrasound-based hyperthermia in cancer therapy.

## 5. Conclusions

Based on the thermal response of biological tissue to a short HIFU sonication, we demonstrated a numerical workflow to compute off-line the prescribed acoustic power yielding an accurate steady-state hyperthermia regimen. Our study supports a novel approach for clinical advancement in ultrasound-based hyperthermia, offering a solution to overcome the logistic burden and regulatory constraints of systematic closed-loop intraoperatory MR control, therefore helping to streamline the deep local hyperthermia procedures in cancer therapy.

## Figures and Tables

**Table 1:** Main parameters of MRgFUS thalamotomy sonications analyzed in this study.

#ID	Age	Skull Density Ratio	Number of Active Elements	Active Surface of Transducer [cm^2^]	Electronic Steering ML, AP, SI [mm]	Measured Acoustic Power [W]	Sonication Duration [s]	Measured Energy [kJ]	Peak Temperature Elevation [°C]
A	76	0.56	933	347.8	0, 0, 0	395.8	12.0	4.38	14.2
B	48	0.45	912	335.7	−1.6, −0.5, 0.5	540.3	8.0	3.81	15.3

**Table 2:** Results for MRgFUS hyperthermia performance (mean and standard deviation of the plateau) using the impulse response formalism targeting +7 °C, applied with different perfusion levels (no perfusion, low perfusion at 4.4 mL/min, and high perfusion at 5.6 mL/min). The drift value represents the average slope of the temperature during the steady-state of approximately 1000 s. TtoSS indicates the time in s to reach 90% of the desired temperature elevation, here 6.3 °C.

Perfusion	N	Mean (°C)	STD (°C)	Drift (10^−3^ °C/s) (Min–Max)	TtoSS (Sampling Points)	TtoSS(s)
no	10	6.94	0.35	0.6 (0.2–1.4)	22	59.4
low	10	7.29	0.32	0.7 (0.3–1.4)	16	43.2
high	10	7.09	0.33	0.9 (0.4–1.6)	15	40.5

**Table 3:** Results for MRgFUS hyperthermia performance (mean and standard deviation of the plateau) using the on-line feedback control [53] targeting +7 °C, applied with different perfusion levels similar to Table 1.

Perfusion	N	Mean (°C)	STD (°C)	Drift (10^−3^ °C/s) (Min–Max)	TtoSS (Sampling Points)	TtoSS (s)
no	5	7.08	0.65	7.0 (1.8–9.6)	68	183.6
low	5	8.03	0.72	8.3 (0.9–13.0)	49	132.3
high	5	8.29	1.06	8.3 (3.6–9.2)	51	140.4

## Data Availability

The original data presented in the study are openly available in Data Green Function at https://doi.org/10.26037/yareta:d7jmcjqd45d5jimn4iiajg2s2i.

## Abbreviations

The following abbreviations are used in this manuscript:

MRgFUS	Magnetic Resonance-guided Focused Ultrasound
HT	Hyperthermia
RT	Radiation Therapy
MR	Magnetic Resonance
MRI	Magnetic Resonance Imaging
SAR	Specific Absorption Rate
HIFU	High-Intensity Focused Ultrasound
GGF	Generalized Gaussian Function
AUC	Area Under Curve
PTFE	Polytetrafluoroethylene
PRFS	Proton Resonance Frequency Shift
GRE	Gradient Echo
EPI	Echo-Planar Imaging
FOV	Field Of View
TR	Repetition Time
TE	Echo Time
BW	Bandwidth
TtoSS	Time to Steady-State
